# Supplementary material for: Relationship between Patient Preferences, Attitudes to Treatment, Adherence, and Quality of Life in New Users of Teriflunomide
Source: Pharmaceuticals (Basel). 2022 Oct 11;15(10):1248. doi: 10.3390/ph15101248 (PMC9609230; doi:10.3390/ph15101248)

Supplementary Table S1. Previous DMT treatments

Previous DMT treatment			No 2nd line	2nd line before teriflunomide				
**1st line before teriflunomide**	**N**	**%**	N	Dacliz.	Glatiram.	I. beta 1a	I. beta 1b	Pegi. beta 1a
Daclizumab	2	4.0%	2	-	-	-	-	-
Glatiramer acetate	17	34.0%	13	-	-	3	1	-
Interferon beta 1a	23	46.0%	17	-	4[1)]	1	-	1
Interferon beta 1b	6	12.0%	3	-	1	2	-	-
Peginterferon beta 1a	2	4.0%	2	-	-	-	-	-
Total	50	100.0%	37	0	5	6	1	1

[1)] One patient had 3 previous DMT treatments; 3rd line was interferon beta 1a.

Supplementary Table S2. Duration of previous DMT treatments

Duration of previous DMT treatment (years)	N	Mean	SD	Median	Minimum	Maximum
**1st line before teriflunomide**	48[1)]	4.7	3.3	4.5	0.0	12.6
Daclizumab	2	5.9	0.9	5.9	5.3	6.6
Glatiramer acetate	17	5.0	3.9	4.7	0.1	11.9
Interferon beta 1a	21[1)]	4.4	3.2	3.9	0.2	12.6
Interferon beta 1b	6	5.5	2.6	6.7	1.0	7.8
Peginterferon beta 1a	2	1.0	1.4	1.0	0.0	2.1
**2nd line before teriflunomide**	12	4.5	3.2	3.7	0.3	10.0
Glatiramer acetate	5	4.9	3.3	4.2	1.4	10.0
Interferon beta 1a	5	5.5	3.0	6.4	1.9	8.7
Interferon beta 1b	1	1.4	-	1.4	1.4	1.4
Peginterferon beta 1a	1	0.3	-	0.3	0.3	0.3
**3rd line before teriflunomide**	1	3.4	-	3.4	3.4	3.4
Interferon beta 1a	1	3.4	-	3.4	3.4	3.4

[1)] Duration is missing for 2 patients.

Supplementary Table S3. The annualized relapse rate after 3 and 9 months of teriflunomide therapy divided by diagnosis and previous treatment[1)]

Annualized relapse rate[2)]	Baseline	Visit 2	Visit 3
**Patients diagnosed with CIS (N = 68)**	-	-	-
**ARR**	0.78 (0.58–1.02)	0.12 (0.05–0.23)	0.15 (0.07–0.27)
**ARR relative decrease**	-	84.9%	81.1%
**Patients diagnosed with RRMS (N = 37)**	-	-	-
**ARR**	0.62 (0.39–0.93)	0.32 (0.17–0.57)	0.16 (0.06–0.35)
**ARR relative decrease**	-	47.8%	73.9%
**DMT naïve patients (N = 55)**	-	-	-
**ARR**	1.15 (0.88–1.47)	0.22 (0.11–0.38)	0.18 (0.09–0.33)
**ARR relative decrease**	-	81.0%	84.1%
**DMT previously treated patients (N = 50)**	-	-	-
**ARR**	0.26 (0.14–0.44)	0.08 (0.02–0.20)	0.12 (0.04–0.26)
**ARR relative decrease**	-	69.2%	53.8%

[1)] Baseline outcomes assessed the period of 12 months before the teriflunomide treatment, at visit 2, a period of 3 months was evaluated (between baseline and visit 2), and at visit 3, a period of 6 months was evaluated (between visit 2 and 3).
[2)] Values are % / mean (95% CI).

Supplementary Table S4. EDSS score distribution (0–10) per visit (N = 105)

EDSS score	Visit 1	Visit 2	Visit 3
0	6.7%	8.6%	4.8%
1	15.2%	11.4%	13.3%
1.5	19.0%	21.0%	23.8%
2	25.7%	25.7%	24.8%
2.5	14.3%	15.2%	14.3%
3	10.5%	7.6%	7.6%
3.5	2.9%	3.8%	3.8%
4	3.8%	4.8%	4.8%
4.5	1.0%	0.0%	1.9%
5	1.0%	1.9%	1.0%

Supplementary Table S5. EDSS score after 3 and 9 months of teriflunomide therapy divided by diagnosis and previous treatment[1)]

EDSS score[2)]	Baseline	Visit 2	Visit 3
**Patients diagnosed with CIS (N = 68)**	1.75 (± 0.88)	1.79 (± 0.95)	1.88 (± 0.91)
**EDSS**	-	-	-
**Patients diagnosed with RRMS (N = 37)**	2.36 (± 1.06)	2.30 (± 1.13)	2.31 (± 1.08)
**EDSS**	-	-	-
**DMT naïve patients (N = 55)**	1.84 (± 0.76)	1.83 (± 0.85)	1.92 (± 0.77)
**EDSS**	-	-	-
**DMT previously treated patients (N = 50)**	2.11 (± 1.18)	2.13 (± 1.20)	2.15 (± 1.18)
**EDSS**	1.75 (± 0.88)	1.79 (± 0.95)	1.88 (± 0.91)

1) Baseline outcomes assessed the 12-month period before teriflunomide treatment, at visit 2, a period of 3 months was evaluated (between baseline and visit 2), and at visit 3 a period of 6 months was evaluated (between visit 2 and 3).
2) Values are mean (± SD).

Supplementary Table S6. The difference in quality of life (MSIS-29) between treatment naïve and previously treated patients at individual visits

Quality of life (MSIS-29)[1)]	Baseline	Visit 2	Visit 3
**Treatment naïve patients (N = 55)**	22.8 (± 19.5)	20.2 (± 16.8)	21.4 (± 17.3)
**Previously treated patients (N = 49)**	30.9 (± 20.8)	31.5 (± 21.9)[3)]	30.1 (± 21.7)
**p-value[2)]**	**0.035**	**0.006**	**0.038**

1) Values are mean (± SD).
2) Two-sample Wilcoxon rank-sum (Mann-Whitney) test, the level of significance was 5% (significant values in bold).
3) N = 50.

Supplementary Figure S1a. Mean quality of life at individual visits (MSIS-29) (N = 104, N = 105, N = 104)

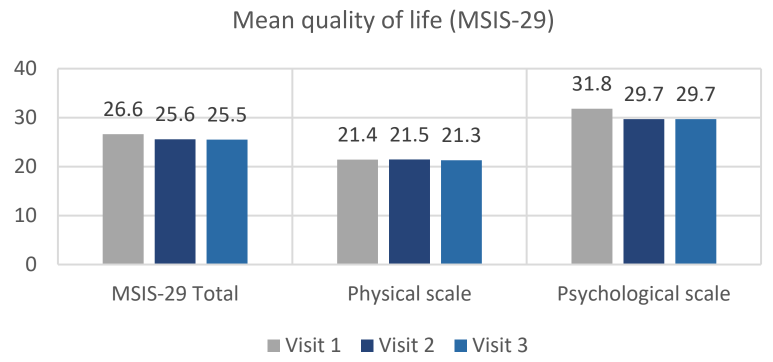

Supplementary Figure S1b. Mean quality of life (MSIS-29) at individual visits by previous treatment (naïve patients N = 55, N = 55, N = 55; previously treated patients N = 49, N = 50, N = 49)

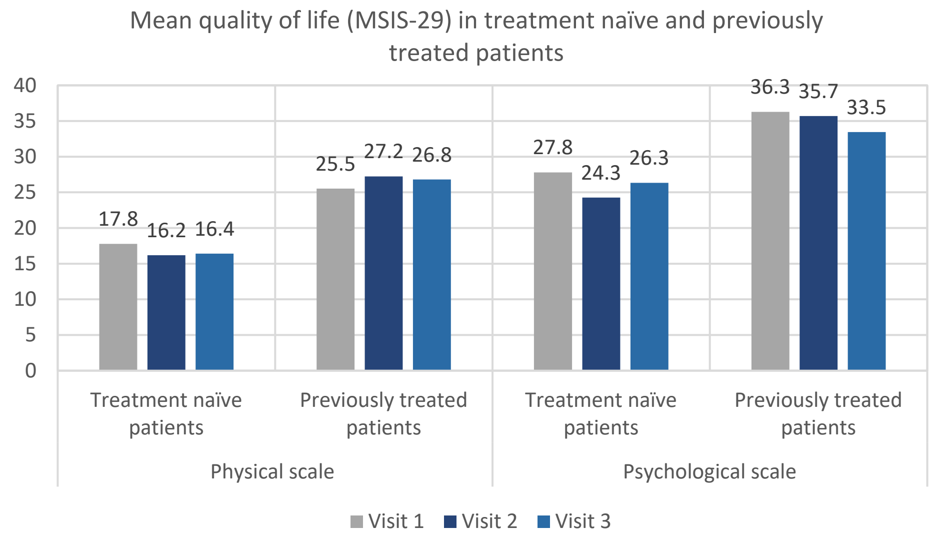

Supplementary Figure S2. Morisky Medication Adherence Scale (MMAS-8) (N = 103, N = 104)

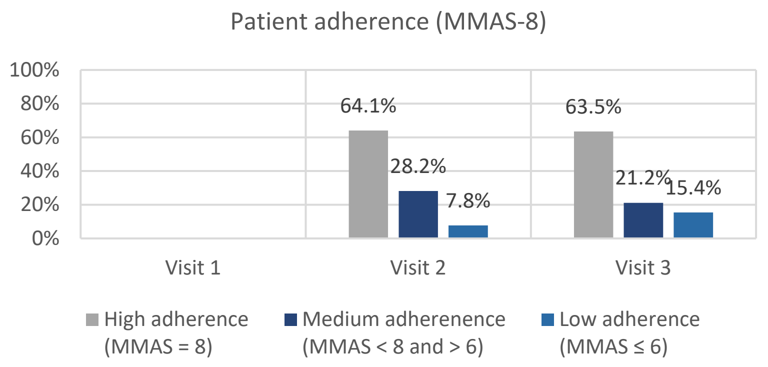

Supplementary Figure S3. Mean quality of life (MSIS-29) at Visit 3; higher MSIS-29 results mean worse quality of life (N = 103)

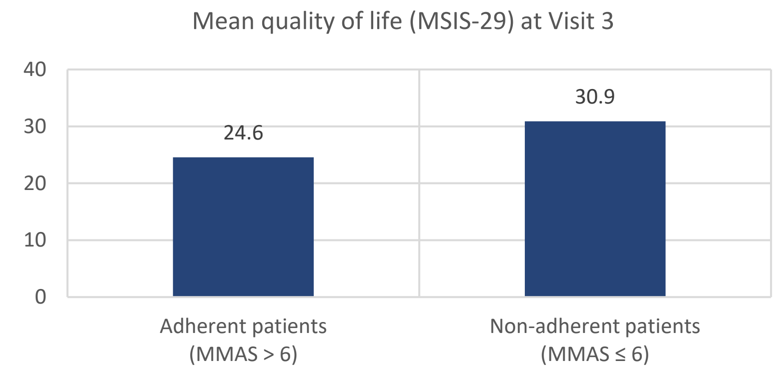

Supplementary Figure S4. Preferred usage form (peroral/injection) (N = 103, N = 104, N = 104)

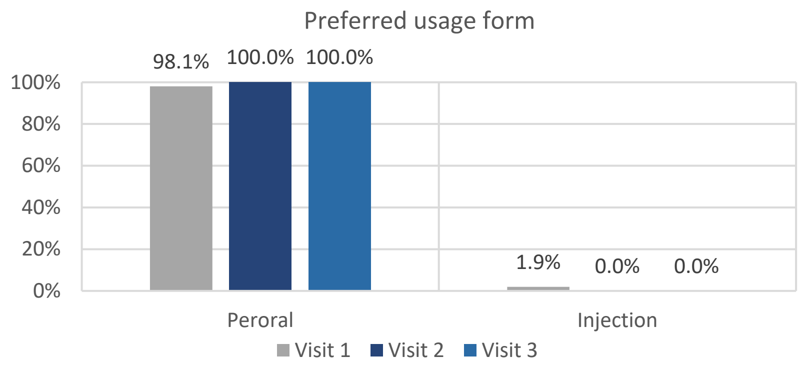

Supplement: Supplementary file 1 [file pharmaceuticals-15-01248-s001.zip › pharmaceuticals-1878067-supplementary.pdf]
